# The Emerging Role of PPAR Beta/Delta in Tumor Angiogenesis

**DOI:** 10.1155/2020/3608315

**Published:** 2020-08-13

**Authors:** Siyue Du, Nicole Wagner, Kay-Dietrich Wagner

**Affiliations:** Université Côte d'Azur, CNRS, INSERM, iBV, 06107 Nice, France

## Abstract

PPARs are ligand-activated transcriptional factors that belong to the nuclear receptor superfamily. Among them, PPAR alpha and PPAR gamma are prone to exert an antiangiogenic effect, whereas PPAR beta/delta has an opposite effect in physiological and pathological conditions. Angiogenesis has been known as a hallmark of cancer, and our recent works also demonstrate that vascular-specific PPAR beta/delta overexpression promotes tumor angiogenesis and progression in vivo. In this review, we will mainly focus on the role of PPAR beta/delta in tumor angiogenesis linked to the tumor microenvironment to further facilitate tumor progression and metastasis. Moreover, the crosstalk between PPAR beta/delta and its downstream key signal molecules involved in tumor angiogenesis will also be discussed, and the network of interplay between them will further be established in the review.

## 1. Introduction

Peroxisome proliferator-activated receptors (PPARs) as ligand-activated transcription factors belong to the steroid receptor superfamily, which includes three isoforms, PPAR alpha, PPAR beta/delta, and PPAR gamma [1]. PPARs form heterodimers with retinoic X receptors and regulate the expression of various genes upon ligand binding. PPARs also interact with corepressors or coactivators to modulate the transcription of its downstream target genes. PPARs as important transcriptional regulators have been suggested to be involved in lipid metabolism and multiple cellular functions. For instance, PPAR alpha also functions in fatty acid beta-oxidation and vascular inflammation [2]. PPAR gamma acts as a regulator in adipocyte differentiation and type 2 diabetes [3]. PPAR beta/delta is a key player in cardiac energy production, angiogenesis, and particularly in cancer progression [4].

PPAR alpha and PPAR gamma exert predominantly an antiangiogenic effect [5–10], but there still exist conflicting studies showing opposite results [11, 12]. On the contrary, PPAR beta/delta produces more obviously proangiogenic effects [13–18]. In this review, we will focus on the promoting role of PPAR beta/delta in angiogenesis, especially in tumor angiogenesis. The network of interplay between PPAR beta/delta and its various downstream signal molecules, and also between those key molecules, will be further discussed and established. Remarkably, diverse important signal molecules involved in tumor angiogenesis and progression, and cancer cell metabolism have been identified as direct PPAR beta/delta target genes.

## 2. Angiogenesis

Angiogenesis is the physiological process through which a new capillary network forms from the preexisting vasculature [19, 20], whereas vasculogenesis denotes de novo blood vessel formation mostly during embryogenesis in which endothelial progenitor cells (EPC) migrate to sites of vascularization, then differentiate into endothelial cells (EC), and coalesce into the initial vascular plexus [21, 22]. Besides the interaction between proangiogenic factors and antiangiogenic factors, angiogenesis is also a multiple step biological process during which a variety of molecules cooperate including cell adhesion molecules, matrix metalloproteinases (MMPs), extracellular matrix (ECM), and basement membrane components.

Angiogenesis is a physiological and vital process in development and growth. An imbalance of proangiogenic and antiangiogenic factors causes angiogenesis in pathological conditions such as diabetic retinopathy and tumor growth. Thus, when the imbalance comes to a point at which angiogenesis is triggered by tumor cells, then an “angiogenic switch” of tumor cells is turned on; during tumor progression, the “angiogenic switch” is often activated and remains on [23–25]. Inducing angiogenesis is known as a hallmark of cancer [26], and angiogenesis is also a fundamental step by which most benign tumors transition into malignant ones.

### 2.1. Tumor Angiogenesis

Tumor needs to sprout new vessels and further develop a vascular network in order to supply nutrients and oxygen, remove waste products, support a continually high proliferative rate, and ultimately expand neoplastic growth [23, 27]. Hence, angiogenesis is essential for helping sustain tumor growth and facilitate tumor progression. Besides being a requirement for angiogenesis, an abnormal vasculature also helps to promote tumor progression and metastasis. The tumor vascular wall is imperfect and prone to leakage, so it is much easier for tumor cells to directly penetrate into the blood vessels or lymphatic vessels and then proliferate at another distant site to form metastasis [28].

Due to intensive abnormal neovascularization in tumor tissues, most malignant tumors grow rapidly and acquire the ability to spread to adjacent and distant organs, which makes them more malignant and even life threatening. Therefore, angiogenesis indeed plays an important role in tumor progression and metastasis, and to intervene with this process would obviously prevent tumor development and spread. Thus, this has been regarded as a critical target for antitumor therapy.

## 3. PPAR Alpha and Angiogenesis

It was reported firstly that a selective PPAR alpha agonist WY14643 did not show any effect on angiogenesis or EC proliferation [29]. But some subsequent studies showed that the activation of PPAR alpha inhibited angiogenesis in vitro by using fenofibrate, a clinically used PPAR alpha agonist [30]. Moreover, fenofibrate suppressed EC proliferation, migration, and tube formation through inhibition of protein kinase B (Akt) and disruption of the cytoskeleton [31]. Furthermore, PPAR alpha activation was shown to inhibit vascular endothelial growth factor- (VEGF-) induced EC migration and basic fibroblast growth factor- (bFGF/FGF2-) induced corneal angiogenesis in vitro and in vivo [5]. Especially, in vivo, reduced tumor growth and microvessel numbers were observed in mice implanted with melanoma, Lewis lung carcinoma (LLC), fibrosarcoma, and glioblastoma due to a systemic treatment of PPAR alpha ligand, and the antiangiogenic state induced through activation of PPAR alpha with elevated thrombospondin-1 (TSP1) and endostatin expression [5].

However, in that same year, it was demonstrated in another observation that activation of PPAR alpha stimulated neovascularization in vivo with increased phosphorylation of endothelial nitric oxide synthase (eNOS) and Akt via a VEGF-dependent manner [32]. Furthermore, Zhang and Ward also suggested that PPAR alpha activation induced proangiogenic responses in human ocular cells [33]. In another study, it was shown that a new PPAR alpha agonist (R)-K-13675 had no effect on angiogenesis [34]. Recently, PPAR alpha activation is further shown to have antineovascularization effects with downregulation of VEGF and angiopoietin expression in a rat alkali burn model [35].

In summary, the role of PPAR alpha in angiogenesis is still controversial. Some observations showed that ligand activation of PPAR alpha had antiangiogenic effects mediated either through upregulation of antiangiogenic factors such as TSP1 and endostatin, or downregulation of proangiogenic factors including VEGF, FGF2, AKT, and angiopoietins. Others also reported opposite results showing a proangiogenic role upon PPAR alpha activation. Thus, the specific molecular mechanism is still unclear and needs to be further studied.

## 4. PPAR Gamma and Angiogenesis

Ligand activation of PPAR gamma was previously shown to inhibit human umbilical vein endothelial cell (HUVEC) tube formation in collagen gels [36] and VEGF-induced choroidal neovascularization in vitro and in vivo [37]. Another study also demonstrated that EC apoptosis was induced through treatment with the PPAR gamma ligand 15d-PGJ2 [38]. Furthermore, rosiglitazone, a potent PPAR gamma agonist, was shown to inhibit primary tumor growth and metastasis through both direct and indirect antiangiogenic effects in vitro, and bFGF-induced corneal neovascularization in vivo [8]. Moreover, a similar observation also displayed the inhibition of VEGF-induced angiogenesis in a chick chorioallantonic membrane model [39]. In a mouse model with ischemia-induced retinopathy, pioglitazone, a PPAR gamma agonist, also showed a protective effect against pathological neoangiogenesis through upregulation of anti-inflammatory adipokine adiponectin [40]. Additionally, the PPAR gamma antagonist GW9662 was shown to reverse Omega-3 polyunsaturated fatty acid-induced reduction of E-Selectin, angiopoietin-2, vascular cell adhesion molecule-1, and intracellular adhesion molecule-1 [41], implicating an antiangiogenic potential of PPAR gamma itself. However, opposite results also showed that pioglitazone enhanced neovascularization and inhibited apoptosis of EPC in vitro and in vivo via a Phosphoinositide-3-Kinase- (PI3K-) dependent manner [42].

Nadra et al. observed that PPAR gamma-null embryos displayed a vascular structural defect at E9.5. Moreover, disorganized placental layers and an altered placental microvasculature were observed in pregnant wild-type mice treated with the PPAR gamma agonist rosiglitazone, as well as reduced expression of proangiogenic factors including VEGF, proliferin, and platelet-endothelial cell adhesion molecule-1 (PECAM1/CD31) [43], suggesting a crucial role of PPAR gamma in placental vascular development. The major antiangiogenic properties on PPAR gamma activation were also reviewed here [44].

Notably, in most cancers, the canonical Wnt/beta-catenin pathway is upregulated, while on the contrary, PPAR gamma is downregulated. Interestingly, in numerous tissues, the activation of PPAR gamma inhibits the beta-catenin pathway, whereas the stimulation of the canonical Wnt/beta-catenin signal cascade also inactivates PPAR gamma [45], implicating a negative regulatory role of PPAR gamma in carcinogenesis where tumor angiogenesis might be a fundamental step.

In summary, PPAR gamma predominantly displays an antiangiogenic effect that may be mediated through the inhibition of VEGF or bFGF-induced neovascularization and reduction of the expression level of some proangiogenic factors.

## 5. PPAR Beta/Delta and Angiogenesis

Unlike PPAR alpha and PPAR gamma, on the contrary, many studies have explicitly shown the proangiogenic effects of PPAR beta/delta on physiological and pathological angiogenesis. The first evidence provided in a study is that activation of PPAR beta/delta with GW501516, a highly selective PPAR beta/delta agonist, induces HUVEC proliferation and an increased expression of VEGF and its receptor VEGFR1 (FLT1) [46]. Besides inducing EC proliferation, PPAR beta/delta activation by its ligand prostacyclin (PGI2) also stimulates upregulation of 14-3-3 alpha expression, an antiapoptotic and anti-inflammatory protein, which thereby protects ECs from H_2_O_2_-induced apoptosis and oxidant injury [47]. Moreover, a subsequent study further provides evidence that activation of PPAR beta/delta with GW501516 induces angiogenesis during which VEGF release is considered as a major trigger factor [48], firstly suggesting the promotion for angiogenesis upon PPAR beta/delta activation.

Müller-Brüsselbach et al. show that PPAR beta/delta -/- mice implanted with LLC and B16 melanoma exhibit diminished blood flow and immature microvascular structures compared with wild-type mice. Moreover, reexpression of PPAR beta/delta into the matrigel-invading cells triggers microvessel maturation and restores normal vascularization [17], indicating a crucial role of PPAR beta/delta in tumor vascularization. Additionally, another study also observed reduced levels of calcium intracellular channel protein 4 (CLIC4), but it observed enhanced expression of cellular retinol binding protein 1 (CRBP1) in migrating ECs from PPAR beta/delta-null mice [49], both of which play a role in tumor vascularization [50, 51]. It was reported that PPAR beta/delta was required for placentation [52], and most of the PPAR beta/delta-null mutant embryos died at E9.5 to E10.5 due to abnormal cell-to-cell communication at the placental-decidual interface [53]. However, in these studies [52–54], a defect in angiogenesis was not observed during normal development in PPAR beta/delta-knockout mice.

Some observations also show the important role of PPAR beta/delta in physiological angiogenesis. For instance, skeletal muscle-specific PPAR beta/delta overexpression leads to an increase in the number of oxidative muscle fibers and running endurance in adult mice [55–57]. Moreover, PPAR beta/delta activation promotes a rapid muscle remodeling via a calcineurin-dependent manner, and induces muscle angiogenesis in highly selective PPAR beta/delta agonist GW0742-treated animals [58]. Furthermore, in the heart, pharmacological PPAR beta/delta stimulation with GW0742 induces rapid cardiac growth and cardiac angiogenesis through direct transcriptional activation of calcineurin [15]. Interestingly, the same cardiac phenotype was also observed after treatment with the PPAR beta/delta agonist GW501516, implicating a response specificity for PPAR beta/delta stimulation [15]. Calcineurin activation further leads to the stimulation of nuclear factor-activated T cell c3 (NFATc3) and an enhanced expression of hypoxia inducible factor 1 alpha (HIF-1alpha) and cyclin-dependent kinase 9 (CDK9) [15]. Overall, the remodeling in skeletal muscle and heart is perfectly the same as the phenotype observed with exercise, and both of them are mediated through activation of calcineurin.

PPAR beta/delta may act as a key regulator in mediating pathological angiogenesis. For instance, PPAR beta/delta was shown to regulate retinal angiogenesis in vitro and in vivo, and its inhibition reduced preretinal neovascularization possibly via an Angiopoietin-like protein 4- (Angptl4-) dependent manner [59], implicating the potential of PPAR beta/delta in modulating pathological ocular angiogenesis. Recently, an observation reported that PPAR beta/delta knockdown in both retinal pigment epithelial and choroidal endothelial cells caused an antiangiogenic phenotype, and PPAR beta/delta promoted laser-induced choroidal neovascular (CNV) lesions in PPAR beta/delta +/+ mice [60]. Moreover, pharmacological inhibition of PPAR beta/delta with the antagonist GSK0660 also resulted in a significantly decreased CNV lesion size in vivo, suggesting a functional role of PPAR beta/delta in the development of CNV lesions [60]. This indicates that PPAR beta/delta has an important association with pathological angiogenesis.

Angiotensin II (Ang II), the biologically active peptide of the renin-angiotensin system (RAS), is a major blood pressure and cardiovascular homeostasis regulator and is also recognized as a potent mitogen. Angiotensin-converting enzyme inhibitors were introduced approximately 30 years ago as antihypertensive agents and have since become a successful therapeutic approach for high blood pressure, congestive heart failure, and postmyocardial infarction. In experimental systems, the antitumor effects of diverse ACE inhibitors show that these inhibit cell proliferation and possess antiangiogenic, antimetastatic, and anti-inflammatory effects [61–63]. It has been shown recently that activation of PPAR beta/delta inhibits Ang II-stimulated protein synthesis in a concentration-dependent manner and suppresses Ang II-induced generation of reactive oxygen species (ROS) in vascular smooth muscle cells [64]. PPAR beta/delta was further shown to inhibit Ang II-mediated atherosclerosis [65]. However, it is not clear until now if PPAR beta/delta activation can be considered as an ACE inhibitor-mimicking approach as it is for example the case for PPAR gamma activators [66]. Furthermore, the relevance of this hypothetical PPAR beta/delta feature might be limited for tumor angiogenesis where vascular smooth muscle hypertrophy and atherosclerosis do not contribute to the major pathology.

Besides inducing angiogenesis, it has been demonstrated that PPAR beta/delta directly acts on early EPC through activation of the AKT pathway and induces an enhanced vasculogenesis [67]. Similarly, the PPAR beta/delta-mediated provasculogenic effects are also observed on late EPC [68]. He et al. showed that PPAR beta/delta activation with GW501516 induced EPC proliferation and tube formation, whereas EPC treated with an inhibitor of cyclooxygenase (COX) or PGI2 synthase, or with PPAR beta/delta-specific siRNA also displayed an opposite effect [68]. Furthermore, it has been demonstrated that PPAR beta/delta induces angiogenesis and skeletal muscle regeneration through matrix metalloproteinase- (MMP-) 9-mediated insulin-like growth factor-1 paracrine networks upon EPC activation [69]. Han et al. also observed that PPAR beta/delta activation promoted a rapid wound healing with enhanced angiogenesis in a mouse model with skin punch wound [69]. Overall, in addition to EC, PPAR beta/delta is also a key regulator of EPC, or even may act as an initiator of activation of EPC to further induce vasculogenesis.

## 6. PPAR Beta/Delta and Tumor Angiogenesis Linked to Tumor Microenvironment

PPAR beta/delta expression is often upregulated and promotes cancer progression in many major human cancers such as colon, lung, breast, and gastric cancers [70–73], which suggests a crucial role of PPAR beta/delta in cancer cells even though there exist some conflicting studies indicating that the functional role of PPAR beta/delta in tumorigenesis or carcinogenesis still remains highly controversial [74–77] and dependent on specific tumor or cancer cell types. Thus, here we discuss the promotion of PPAR beta/delta in tumor progression through facilitating tumor angiogenesis.

PPAR beta/delta has been suggested as a critical “hub node” transcriptional factor which governs a tumor “angiogenic switch” [13, 78–80]. In the transcriptional network analysis, it was reported that tumor growth and tumor angiogenesis were markedly inhibited in PPAR beta/delta-null mice in comparison with wild-type mice [13]. Moreover, the elevated PPAR beta/delta expression level was also considered to be highly correlated to pathologically advanced tumor stage and increased cancer risk for recurrence and distant metastasis in patients with pancreatic cancer [13], indicating the crucial association of PPAR beta/delta with tumor angiogenesis, progression, and cancer invasiveness.

PPAR beta/delta may indirectly facilitate tumor angiogenesis and progression through its function on the tumor microenvironment (TME) where tumor angiogenesis is fostered. Moreover, a tumor also releases some extracellular signals to closely communicate and constantly collaborate with TME to facilitate tumor angiogenesis, in order to further enable tumor growth and progression. For instance, it was shown that colon cancer cells with PPAR beta/delta knockout failed to stimulate EC vascularization in response to hypoxic stress, whereas wild-type cells exposed to hypoxia were able to induce angiogenesis [81, 82], suggesting that PPAR beta/delta is required for the promotion of angiogenesis in hypoxic stress-mediated TME. Moreover, in the TME, tumor-infiltrating myeloid cells are considered as the most important cells for fostering tumor angiogenesis among the multiple different kinds of stromal cells [82]. Besides stimulating tumor angiogenesis, tumor myeloid cells also support tumor growth by suppressing tumor immunity and promoting tumor metastasis to distinct sites [83]. Interestingly, it has been demonstrated that PPAR beta/delta activation in tumor-infiltrating myeloid cells stimulates cancer cell invasion and facilitates tumor angiogenesis via an Interleukin 10- (IL10-) dependent manner [84]. Moreover, impaired tumor growth and angiogenesis were observed in PPAR beta/delta KO BMT mice due to PPAR beta/delta deficiency in tumor myeloid cells [84], suggesting that PPAR beta/delta plays a key role in tumor angiogenesis and progression in tumor myeloid cells of TME.

Furthermore, the endoplasmic reticulum (ER), an essential organelle involved in many cellular functions, is implicated in TME. In cancer, stressors like hypoxia, nutrient deprivation, and acidosis disrupt ER function and lead to accumulation of unfolded proteins in ER, a condition known as ER stress. Cells adapt to ER stress by activating an integrated signal transduction pathway called the unfolded protein response (UPR). UPR represents a survival response by the cells to restore ER homeostasis and has both survival and cell death effects. The mechanisms that determine cell fate during ER stress are not well understood. For instance, short exposure to ER stress initially increases AKT signaling, but long-term ER stress suppresses AKT signaling [85]. PPAR beta/delta activation has been shown to reduce endoplasmic reticulum (ER) stress-associated inflammation in skeletal muscle through an AMPK-dependent mechanism [86] and to reduce inflammation in response to chronic ER stress in cardiac cells [87]. Furthermore, it has been nicely shown that PPAR beta/delta can repress RAS-oncogene-induced ER stress to promote senescence in tumors [88] This is mediated through the decrease of p-AKT activity promoting cellular senescence through upregulation of p53 and p27 expression [89]. It would be interesting to investigate the direct effects of PPAR beta/delta on senescence of tumor endothelial cells in an in vivo setting. We recently showed that senescent endothelial cells are indispensable for a healthy lifespan and that removal of senescent endothelium disrupts vascular function leading to diminished vessel densities and fibrotic lesions [90]. If PPAR beta/delta mediates senescence of tumor endothelium thereby protecting vessel integrity, this might explain the enhanced tumor growth and vascularization upon PPAR beta/delta activation observed by us and others [13, 16, 77].

Most recently, Zuo et al. demonstrated that PPAR beta/delta in cancer cells regulates tumor angiogenesis in vivo and in vitro by promoting the secretion of proangiogenic factors including VEGF and Interleukin 8 (IL8) [18]. Most importantly, in our recent works, it has been shown that conditional inducible vascular endothelium-specific PPAR beta/delta overexpression in vivo leads to enhanced tumor angiogenesis, tumor growth, and metastasis formation, further indicating a vascular EC-specific PPAR beta/delta action mechanism in tumor progression, independent of some controversial observations of PPAR beta/delta in specific tumor or cancer cell types [16]. Wagner et al. also firstly reported the mouse model in which rapid induction of cardiac angiogenesis and cardiac hypertrophy were observed [91, 92].

### 6.1. Crosstalk between PPAR Beta/Delta and Signal Molecules

PPAR beta/delta activation or overexpression may upregulate the expression of its various downstream signal molecules involved in tumor angiogenesis including proangiogenic factors (such as VEGF, PDGF, and FGF), proinvasive matrix-degrading enzymes (such as MMP9), proinflammatory mediators (such as COX2), and cytokines and chemokines (such as IL1 and CXCL8), even some of which have been further identified as PPAR beta/delta direct target genes. Besides a leading role of PPAR beta/delta among the signal molecules, PPAR beta/delta may function in TME linked to diverse kinds of cells through direct or indirect modulation of its downstream molecules.

#### 6.1.1. Interplay between PPAR Beta/Delta and Inflammatory Angiogenesis

Inflammatory angiogenesis is a crucial process in tumor progression. For instance, the proinflammatory mediator cyclooxygenase-2 (COX2) is considered as a key regulator of angiogenesis and tumor growth through multiple downstream proangiogenic mechanisms such as production of VEGF and induction of MMPs. Moreover, selective inhibition of COX2 has also been shown to suppress angiogenesis in vivo and in vitro [93]. It is well known that VEGFA plays a critical role in both angiogenesis and vasculogenesis [94], and it leads the directional migration of tip cells and stalk cell proliferation in microtubule branches [95, 96]. It has also been demonstrated that MMP9 triggers the “angiogenic switch” during carcinogenesis and enhances the availability of VEGF to its receptors [97]. Furthermore, it has been reported that inflammatory cell MMP9 initiates the onset of tumor neovascularization during which there exists functional links between VEGF and MMPs including MMP9 [98]. LEPTIN is shown to mediate angiogenesis in vivo and in vitro through induction of EC proliferation and expression of MMP2 and MMP9 [99], and to further promote EC differentiation and directional migration through enhancement of COX2 activity [100]. LEPTIN could also induce angiogenesis via transactivation of VEGFR in ECs [101]. Additionally, besides inducing angiogenesis, PPAR beta/delta also functions in chronic inflammation-facilitating tumorigenesis through induction of COX2 and its product prostaglandin E2 (PGE2) in vivo [102, 103]. Interestingly, COX2, VEGF, MMP9, and LEPTIN have been identified as PPAR beta/delta target genes via a direct transcriptional activation mechanism in hepatocellular carcinoma cells [104], colorectal cancer cells [105, 106], EPCs [67, 69], and liposarcoma cells [107], respectively.

In TME, tumor-infiltrating inflammatory cells also help to induce and sustain tumor angiogenesis, and further to facilitate tissue invasion and tumor metastatic spread by releasing some signal molecules such as proinvasive MMP9 and inflammatory chemokines [108–110]. Chemotaxis is also a crucial process for inducing angiogenesis in tumors, either directly by attracting ECs towards tumor cells to form new vessels, or indirectly by mediating immune inflammatory cells to infiltrate, eventually promoting tumor angiogenesis [111]. Chemotaxis of tumor cells and stromal cells in TME is also required for tumor dissemination during tumor progression and metastasis [110, 111].

CXC chemokines such as CXCL8 (encoding IL8) and CXCL5 are also involved in COX2-associated angiogenesis to contribute to non-small-cell lung cancer progression [111, 112]. It is further shown that IL8 directly regulates angiogenesis via recruitment of neutrophils [112], which further drives VEGF activation [113]. Moreover, IL8-responding neutrophils are considered as the major source of angiogenesis-inducing MMP9 [98, 114]. Chemokine C-C motif ligand 2 (CCL2), in addition to the promotion of angiogenesis [115, 116], also enhances tumor metastasis [117]. Furthermore, myeloid monocytic cells such as myeloid-derived suppressor cells (MDSCs), tumor-associated macrophages (TAMs), and dendritic cells are recruited to the tumor site mainly by CCL2 and produce many proangiogenic factors such as VEGF, CXCL8, platelet-derived growth factor (PDGF), and transforming growth factor beta (TGF beta) [118–120]. In fact, both TGF beta and hypoxia are potent inducers of VEGF expression in tumor cells and collaborate with TME to provide the foundation of tumor angiogenesis and cancer cell invasion [121]. Importantly, IL8 has been reported as a key target gene of PPAR beta/delta to promote angiogenesis in vivo and in vitro [18], and CCL2 expression is also significantly upregulated upon vascular PPAR beta/delta overexpression in vivo [16].

COX2 also mediates IL1 beta-induced angiogenesis in vitro and in vivo [122, 123]. IL1 beta supports neovascularization through the regulation of the expression of VEGF and its receptor VEGFR2 (FLK1/KDR) on ECs [124]. IL1 acts as an upstream proinflammatory mediator that initiates and disseminates the inflammatory state by inducing a local interactive network and increasing adhesion molecule expression on ECs and leukocytes, which facilitates tumor-associated angiogenesis [125]. In TME, inflammatory IL1 beta recruits myeloid cells from bone marrow and activates them to produce proangiogenic factors such as VEGF; VEGF further activates ECs and myeloid cells, promoting tumor invasiveness and fostering tumor angiogenesis [125]. In addition, IL6 also stimulates angiogenesis and vasculogenesis [126, 127]. However, Gopinathan et al. observed an IL6-induced newly forming vascular structure with defective pericyte (PC) coverage ex vivo [128], thus facilitating cancer cell infiltration and tumor metastasis through vascular leakage. Interestingly, IL1 and IL6 expression levels are significantly upregulated in the PPAR beta/delta overexpression mouse model reported recently [16].

In summary, PPAR beta/delta seems to act as a key leader in inflammatory mediator-driven tumor angiogenesis linked to TME in which many proinflammatory mediators, chemokines, and proangiogenic factors closely communicate with each other, and also associate with tumor-infiltrating myeloid cells such as neutrophils, TAMs, and MDSCs.

#### 6.1.2. Other Key PPAR Beta/Delta-Mediated Proangiogenic Factors

It has been demonstrated that Wilms' tumor suppressor WT1 is a major regulator of tumor neovascularization and tumor progression [129]. E26 avian leukemia oncogene 1 (ETS1) also plays a key role in regulating vascular development and haemopoiesis, particularly in angiogenesis [130]. In addition, ETS1 promotes cancer cell invasion through upregulation of MMPs [131]. Consistent with this, silencing of ETS1 in highly invasive breast cancer cells also reduces the expression of MMP9 and MMP1 [132].

ETS1 also acts as a key regulator of MMPs such as MMP1, MMP3, and MMP9 in human cancer-associated fibroblasts (CAFs) [133, 134]. CAFs support tumor growth by secreting growth factors such as VEGF, FGF, PDGF, and chemokines to stimulate angiogenesis and thereby promote cancer cell invasion and metastasis formation [135, 136]. CAFs, as metastatic tumor stroma, are a crucial component in tumor progression through the remodeling of the ECM structure, thus helping a tumor to acquire an aggressive phenotype [136, 137]. PPAR beta/delta in CAFs also exhibits a protumorigenic effect. It was reported that ablation of PPAR beta/delta in CAFs attenuated tumor growth by altering the redox balance in TME [138], suggesting that PPAR beta/delta in CAFs is also an important player in tumor development. ETS1 induces the expression of VEGF, VEGFR1, and VEGFR2 in ECs [139–141]. In turn, VEGF is also a major inducer of ETS1 in ECs through the activation of either the PI3K/AKT pathway or the MEK/ERK/1/2 signal cascade [142, 143]. WT1 is also reported to regulate tumor angiogenesis via direct transactivation of ETS1 [144].

SRY-related HMG-box 18 (SOX18) has also been reported previously to induce angiogenesis during tissue repair and wound healing [145] and cancer progression [146]. And most recently, it was further shown that specific EC-derived endovascular progenitors initiated a vasculogenic process and differentiated into more mature endothelial phenotypes within the core of the growing tumors through reactivation of SOX18 [147]. Interestingly, these important proangiogenic molecules including WT1, ETS1, and SOX18 are also significantly upregulated in the vascular PPAR beta/delta overexpression model in vivo [16]. And, WT1 is also identified as a target gene of PPAR beta/delta in melanoma cells [148].

#### 6.1.3. PPAR Beta/Delta May Facilitate Cancer Progression at Diverse Cellular Levels in TME

PPAR beta/delta activation is shown to induce colonic cancer stem cell (CSC) expansion and to promote the liver metastasis of colorectal cancer in vivo via direct transactivation of the Nanog gene [149]. NANOG as a key transcriptional factor governs the self-renewal and pluripotency of stem cells [150], and cancer cells expressing NANOG also often exhibit stem cell properties [151]. Protooncogene c-KIT/CD117 is known as the mast/stem cell factor receptor and receptor tyrosine kinase, and its activation in CSCs may regulate the stemness to control tumor progression and drug resistance to tyrosine kinase inhibitors. Moreover, c-KIT has been identified as a potential marker of the cancer stem-like cells [152]. In addition, c-KIT not only functions on ECs [153, 154] but also belongs to the tumor angiogenesis-promoting molecule [155–158]. Studies also suggested that activation of c-KIT enhances the expression of VEGF that can be suppressed by imatinib, an inhibitor of c-KIT in gastrointestinal stromal tumor cells, which thereby has an impact on tumor angiogenesis [159, 160]. c-KIT is also involved in pathological ocular neovascularization [161] and is regulated transcriptionally by WT1 [129] and PPAR beta/delta [16].

PDGFB and its receptor PDGFR beta, also known as angiogenic factors, are suggested to enhance angiogenesis and vasculogenesis via their function in ECs [162–164] and EPCs [165], and to regulate vascular permeability and vessel maturation through recruitment of pericytes (PCs) [166, 167] and smooth muscle cells (SMCs) [168] in newly forming vessels. Moreover, PDGFB and PDGFR beta also interact with other proangiogenic factors such as FGF2 [169, 170], VEGFA, and its receptor VEGFR2 [163]. Furthermore, PDGFB and PDGFR beta may also affect cancer growth and progression by directly acting on TME. Besides the crosstalk with CAFs [171–173], PDGFR beta in stromal fibroblasts may mediate PDGFB-induced TAM recruitment [174], thus implicating a role of PDGFR beta in tumor stroma to facilitate tumor progression. Most recently, it was further shown that specific targeting of PDGFR beta kinase activity in TME inhibited cancer growth and vascularization in cancers with high PDGFB expression such as LLC [175]. Therefore, this indicates the diverse role of PDGFB and PDGFR beta in facilitating tumor angiogenesis and progression at different cellular levels in TME. PDGFR beta is demonstrated as a target of telomeric repeat binding factor 2 (TRF2) that is further activated transcriptionally by WT1 [176]. PDGFB and PDGFR beta have further been identified as critical targets of PPAR beta/delta via a direct transactivation mechanism in vivo [16].

**Figure 1:**
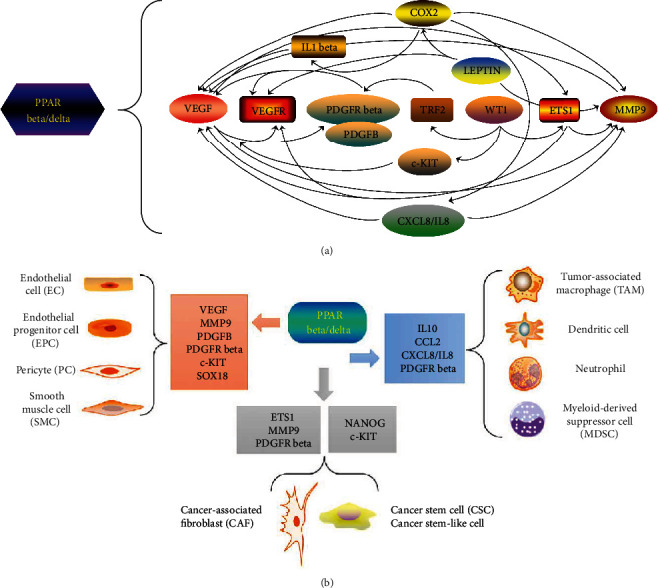
The “hallmark” role of PPAR beta/delta in tumor angiogenesis and progression. (a) Interplay between PPAR beta/delta and downstream key signal molecules. In the signal network of proangiogenic molecules, COX2 promotes the secretion of VEGF and MMPs including MMP9; COX2 infiltration also mediates IL1 beta-induced angiogenesis, which further activates VEGF; COX2 also contributes to cancer progression through the enhancement of the angiogenic chemokine CXCL8 (IL8) expression. IL8 drives VEGF activation and induces MMP9 expression. LEPTIN induces MMP9 expression, enhances COX2 activity, or transactivates VEGFR to facilitate angiogenesis. WT1 transactivates ETS1, TRF2, and c-KIT. ETS1 further upregulates the MMP9, VEGF, and VEGFR expression. In turn, VEGF is also a major inducer of ETS1. TRF2 transactivates PDGFR beta, and c-KIT may affect angiogenesis through the promotion of VEGF production. There exists a crosstalk between VEGF and MMP9 and between VEGF, VEGFR, and PDGFB and PDGFR beta. Oval shape: they represent those molecules that have been identified as direct target genes of PPAR beta/delta; rectangle shape: they represent those molecules that are significantly upregulated upon PPAR beta/delta overexpression. (b) Function of PPAR beta/delta at diverse cellular levels in TME. In TME, multiple distinct cells communicate and collaborate to enable tumor growth and progression. These cells include cancer cells, CSCs, ECs, EPCs, PCs, SMCs, CAFs, and tumor-infiltrating inflammatory cells. PPAR beta/delta can directly function on ECs and EPCs, or directly take action on them by regulating downstream signal molecules such as VEGF, MMP9, PDGFB, PDGFR beta, and SOX18. PDGFB and PDGFR beta regulate vascular permeability and maturation through the recruitment of PCs and SMCs. c-KIT also functions on ECs, c-KIT, and NANOG and may regulate the stemness to control cancer progression. ETS1 regulates MMP9 expression in CAFs; the crosstalk between CAFs and PDGFR beta also exists. PPAR beta/delta may act on tumor-infiltrating myeloid cells through the modulation of the IL10, IL8, CCL2, and PDGFR beta expression. As mentioned above, PPAR beta/delta stimulates cancer cell invasion and facilitates tumor angiogenesis in an IL10-dependent manner in tumor-infiltrating myeloid cells. IL8 can directly regulate angiogenesis via the recruitment of neutrophils. CCL2 is also a major regulator of recruitment of the myeloid monocytic cells such as MDSCs, TAMs, and dendritic cells. Also, PDGFR beta in stromal fibroblasts may mediate PDGFB-induced TAM recruitment. Among these molecules, SOX18, IL10, CCL2, and ETS1 are overexpressed upon PPAR beta/delta activation, and the others have been reported as direct targets of PPAR beta/delta.

In conclusion, a variety of key signal molecules involved in tumor angiogenesis and tumor progression and metastasis have either been identified as PPAR beta/delta direct targets or largely upregulated in the vascular PPAR beta/delta overexpression model in vivo reported recently [16]. Thus, PPAR beta/delta activation seems to give rise to a highly angiogenic phenotype, and even plays a “hallmark” role in promoting tumor angiogenesis and progression. Interestingly, it appears that there could also exist a widely interactive network between the downstream protumor-angiogenic molecules as described above. Therefore, the crosstalk network is established between PPAR beta/delta and the various signal molecules, and also between those molecules (Figure 1(a)).

Moreover, in addition to cancer cells, PPAR beta/delta may also produce pleiotropic effects in TME by modulating downstream key molecules to act on ECs, EPCs, PCs, SMCs, CSCs, CAFs, and tumor-infiltrating inflammatory cells, indirectly facilitating tumor angiogenesis and further promoting cancer development (Figure 1(b)).

### 6.2. Other PPAR Beta/Delta Target Genes

PPAR beta/delta regulates the transcription of target genes via a direct PPRE-dependent transactivation mechanism. The peroxisome proliferator response element (PPRE) comprises a direct repeat (DR) of AGGTCA separated by one nucleotide (DR1) as AGGTCA (N) AGGTCA [177]. But currently, it was shown that only PPAR alpha binds to this sequence; whether ligand activation has an impact on PPARs binding to DNA response elements is still controversial [4]. A variety of genes have been identified as direct targets of PPAR beta/delta and are known to be involved in various cellular biological processes such as fatty acid oxidation, cell survival, inflammation, angiogenesis, cancer cell metabolism, and tumor progression. Direct target genes of PPAR beta/delta identified to date already include Calcineurin A, COX2, VEGF, MMP9, LEPTIN, IL8, WT1, NANOG, c-KIT, PDGFB, PDGFRB, ANGPTL4, PDK4, FABP4, CDKN1C, SRC, EDG2, FOXO1, GLUT1, and SLC1-A5 (Table 1).

As mentioned above, most of these PPAR beta/delta target genes have been suggested to be involved in tumor angiogenesis and progression. ANGPTL4 is a well-known target gene of PPAR beta/delta [183, 184], and it promotes angiogenesis [178, 179], cancer cell invasion [180], and tumor progression and metastasis [181, 182]. Pyruvate dehydrogenase kinase 4 (PDK4) may promote cancer progression by regulating epithelial-mesenchymal transition (EMT) [185, 186] and cancer cell metabolism [186–188]. Fatty acid binding protein 4 (FABP4) may affect cell proliferation and apoptosis by regulating glucose and lipid metabolism [190, 191]. Both PDK4 and FABP4 are the established targets of PPAR beta/delta respectively [189, 192].

Cyclin-dependent kinase inhibitor 1C (CDKN1C) gene, which encodes the cell cycle inhibitor p57^KIP2^, has been suggested to be involved in the regulation of several cancer hallmarks such as inducing angiogenesis, and it has been tested as a prognostic factor for various cancers [17, 193], as well as a target of PPAR beta/delta [17]. Oncogene SRC has been reported to be a direct PPAR beta/delta target, and its tyrosine kinase activity triggers the EGFR/ERK1/2 signal cascade, which promotes the development of ultraviolet radiation-induced skin cancer [194]. Endothelial differentiation gene 2 (EDG2) is also transactivated directly by PPAR beta/delta in late EPCs and leads to enhanced vasculogenesis [195]. Forkhead box protein O1 (FOXO1) is required for EC proliferation and vascular growth [196, 198], and directly regulates VEGFA expression during wound healing [197]. In addition to the physiological angiogenesis, FOXO1 is suggested to be involved in developmental and pathological angiogenesis [198], which is also activated transcriptionally by PPAR beta/delta [199].

Finally, glucose transporter 1 (GLUT1/SLC2A1), as a member of the GLUT family, is widely expressed in many types of cancer cells and plays a key role in glucose uptake for cancer cell metabolism to enable tumor cell growth and proliferation [200, 201]. Neutral amino acid transporter B (SLC1-A5) is an important glutamine transporter in the regulation of essential amino acid influx [203]; and importantly, depletion of SLC1-A5 is demonstrated to abolish tumor progression [204]. Both GLUT1 and SLC1-A5 have been suggested to facilitate tumor progression and are transactivated directly by PPAR beta/delta [202].

For further information, PPARbeta/delta-related signaling pathways are covered by KEGG (Kyoto Encyclopedia of Genes and Genomes) (PATHWAY: map 03320), by the REACTOME pathway database (R-HSA-446176), and by the Protein-Protein Interaction Networks Functional Enrichment Analysis in STRING functional protein association network database (https://string-db.org/cgi/network.pl?taskId=OUdxEiHw19dW).

## 7. Conclusion

PPAR alpha and PPAR gamma seem to have an antiangiogenic role, but there are still conflicting observations. Unlike them, PPAR beta/delta exerts proangiogenic effects. Especially, there exists an intensive crosstalk between PPAR beta/delta and various signal molecules including the identified target genes, and also between those molecules. PPAR beta/delta plays a leading role in the network of interplay by directly and indirectly modulating the downstream proinflammatory or protumorigenic angiogenic molecules which further act on multiple different cell types in TME, thus indicating a potent “hallmark” role of PPAR beta/delta in tumor angiogenesis, cancer progression, and metastasis.

## Figures and Tables

**Table 1:** List of target genes of PPAR beta/delta.

PPAR beta/delta target genes	Cellular biological function	References (for target genes)
Calcineurin A	Induction of cardiac vascularization, cardiac growth, and skeletal muscle remodeling [47, 48]	[15]
COX2	An inflammatory angiogenic mediator and a key regulator of tumor angiogenesis [93, 122, 123]	[104]
VEGF	A key regulator of vasculogenesis and angiogenesis [74, 75, 76]	[105, 106]
MMP9	A proinvasive matrix-degrading enzyme and a key regulator of tumor angiogenesis and metastasis [77, 78]	[69]
LEPTIN	Regulation of endothelial cell behavior and angiogenesis [79, 80, 81]	[107]
IL8	A key angiogenic chemokine, a proinflammatory mediator, and a key regulator of tumor angiogenesis and progression [98, 111, 112, 114]	[18]
WT1	An important regulator of tumor angiogenesis and progression [129]	[148]
NANOG	Regulation of self-renewal of cancer stem cells or cancer stem-like cells [149–151]	[149]
c-KIT	A potential marker of cancer stem-like cells [152]; promotion of tumor angiogenesis [155–158] and pathological ocular neovascularization [161]	[16]
PDGFB	A key regulator of angiogenesis and vasculogenesis [162–165] and vascular permeability and maturation [166–168]	[16]
PDGFR beta	A key regulator of angiogenesis and vasculogenesis [162–165], vascular permeability and maturation [166–168], and tumor progression [174, 175]	[16]
ANGPTL4	Promotion of angiogenesis, tumor progression, and metastasis [178–182]	[183, 184]
PDK4	Regulation of EMT and cell metabolism, and cancer progression [185–188]	[189]
FABP4	Regulation of glucose and lipid metabolism; cell proliferation and apoptosis [190, 191]	[192]
CDKN1C	A prognostic factor for many types of cancer; regulation of angiogenesis and cancer hallmarks [193]	[17, 46]
SRC	Promotion of angiogenesis, cancer invasion, and tumor progression [194]	[194]
EDG2	Enhancement of endothelial cell differentiation and vasculogenesis [195]	[195]
FOXO1	Involvement of physiological, pathological, and developmental angiogenesis [196–198]	[199]
GLUT1	Promotion of cancer cell metabolism and tumor growth [200, 201]	[202]
SLC1-A5	Promotion of cancer cell metabolism and tumor progression [203, 204]	[202]

## References

[1] Moraes L. A., Piqueras L., Bishop-Bailey D. (2006). Peroxisome proliferator-activated receptors and inflammation. *Pharmacology & Therapeutics*.

[2] van Raalte D. H., Li M., Pritchard P. H., Wasan K. M. (2004). Peroxisome proliferator-activated receptor (PPAR)-: a pharmacological target with a promising future. *Pharmaceutical Research*.

[3] Ahmadian M., Suh J. M., Hah N. (2013). PPAR*γ* signaling and metabolism: the good, the bad and the future. *Nature Medicine*.

[4] Wagner K. D., Wagner N. (2010). Peroxisome proliferator-activated receptor beta/delta (PPAR*β*/*δ*) acts as regulator of metabolism linked to multiple cellular functions. *Pharmacology & Therapeutics*.

[5] Panigrahy D., Kaipainen A., Huang S. PPARalpha agonist fenofibrate suppresses tumor growth through direct and indirect angiogenesis inhibition.

[6] Yokoyama Y., Xin B., Shigeto T. (2007). Clofibric acid, a peroxisome proliferator-activated receptor alpha ligand, inhibits growth of human ovarian cancer. *Molecular Cancer Therapeutics*.

[7] Pozzi A., Capdevila J. H. (2008). PPAR Ligands as Antitumorigenic and Antiangiogenic Agents. *PPAR Research*.

[8] Panigrahy D., Singer S., Shen L. Q. (2002). PPARgamma ligands inhibit primary tumor growth and metastasis by inhibiting angiogenesis. *Journal of Clinical Investigation*.

[9] Keshamouni V. G., Arenberg D. A., Reddy R. C., Newstead M. J., Anthwal S., Standiford T. J. (2005). PPAR-*γ* Activation Inhibits Angiogenesis by Blocking ELR+CXC Chemokine Production in Non-small Cell Lung Cancer. *Neoplasia*.

[10] Copland J. A., Marlow L. A., Kurakata S. (2006). Novel high-affinity PPAR _*γ*_ agonist alone and in combination with paclitaxel inhibits human anaplastic thyroid carcinoma tumor growth via p21^WAF1/CIP1^. *Oncogene*.

[11] Kaipainen A., Kieran M. W., Huang S. (2007). PPARalpha deficiency in inflammatory cells suppresses tumor growth. *PLoS ONE*.

[12] Tian L., Zhou J., Casimiro M. C. (2009). Activating peroxisome proliferator-activated receptor gamma mutant promotes tumor growth in vivo by enhancing angiogenesis. *Cancer Research*.

[13] Abdollahi A., Schwager C., Kleeff J. Transcriptional network governing the angiogenic switch in human pancreatic cancer.

[14] Yoshinaga M., Kitamura Y., Chaen T. (2009). The simultaneous expression of peroxisome proliferator-activated receptor delta and cyclooxygenase-2 may enhance angiogenesis and tumor venous invasion in tissues of colorectal cancers. *Digestive Diseases and Sciences*.

[15] Wagner N., Jehl-Piétri C., Lopez P. (2009). Peroxisome proliferator-activated receptor beta stimulation induces rapid cardiac growth and angiogenesis via direct activation of calcineurin. *Cardiovascular Research*.

[16] Wagner K. D., Du S., Martin L., Leccia N., Michiels J. F., Wagner N. (2019). Vascular PPAR*β*/*δ* promotes tumor angiogenesis and progression. *Cells*.

[17] Müller-Brüsselbach S., Kömhoff M., Rieck M. (2007). Deregulation of tumor angiogenesis and blockade of tumor growth in PPARbeta-deficient mice. *The EMBO Journal*.

[18] Zuo X., Xu W., Xu M. (2017). Metastasis regulation by PPARD expression in cancer cells. *JCI Insight*.

[19] Carmeliet P. (2005). VEGF as a Key Mediator of Angiogenesis in Cancer. *Oncology*.

[20] Carmeliet P., Jain R. K. (2011). Molecular mechanisms and clinical applications of angiogenesis. *Nature*.

[21] Carmeliet P. (2003). Angiogenesis in health and disease. *Nature Medicine*.

[22] Risau W., Flamme I. (1995). Vasculogenesis. *Annual Review of Cell and Developmental Biology*.

[23] Hanahan D., Folkman J. (1996). Patterns and emerging mechanisms of the angiogenic switch during tumorigenesis. *Cell*.

[24] Bergers G., Benjamin L. E. (2003). Tumorigenesis and the angiogenic switch. *Nature Reviews Cancer*.

[25] Baeriswyl V., Christofori G. (2009). The angiogenic switch in carcinogenesis. *Seminars in Cancer Biology*.

[26] Hanahan D., Weinberg R. A. (2011). Hallmarks of cancer: the next generation. *Cell*.

[27] McDougall S. R., Anderson A. R. A., Chaplain M. A. J. (2006). Mathematical modelling of dynamic adaptive tumour-induced angiogenesis: clinical implications and therapeutic targeting strategies. *Journal of Theoretical Biology*.

[28] Sherwood L. M., Parris E. E., Folkman J. (1971). Tumor angiogenesis: therapeutic implications. *New England Journal of Medicine*.

[29] Marx N., Sukhova G. K., Collins T., Libby P., Plutzky J. (1999). PPARalpha activators inhibit cytokine-induced vascular cell adhesion molecule-1 expression in human endothelial cells. *Circulation*.

[30] Pille J.-V., Varet J., Vincent L. (2003). Fenofibrate inhibits angiogenesis in vitro and in vivo. *Cellular and Molecular Life Sciences (CMLS)*.

[31] Varet J., Douglas S. K., Gilmartin L. (2010). VEGF in the lung: a role for novel isoforms. *American Journal of Physiology-Lung Cellular and Molecular Physiology*.

[32] Biscetti F., Gaetani E., Flex A. (2008). Selective activation of peroxisome proliferator-activated receptor (PPAR) alpha and PPAR gamma induces neoangiogenesis through a vascular endothelial growth factor-dependent mechanism. *Diabetes*.

[33] Zhang J. Z., Ward K. W. (2010). WY-14 643, a selective PPAR{alpha} agonist, induces proinflammatory and proangiogenic responses in human ocular cells. *International Journal of Toxicology*.

[34] Kitajima K., Miura S., Mastuo Y., Uehara Y., Saku K. (2009). Newly developed PPAR-*α* agonist (R)-K-13675 inhibits the secretion of inflammatory markers without affecting cell proliferation or tube formation. *Atherosclerosis*.

[35] Arima T., Uchiyama M., Nakano Y. (2017). Peroxisome proliferator-activated receptor alpha agonist suppresses neovascularization by reducing both vascular endothelial growth factor and angiopoietin-2 in corneal alkali burn. *Scientific Reports*.

[36] Xin X., Yang S., Kowalski J., Gerritsen M. E. (1999). Peroxisome proliferator-activated receptor gamma ligands are potent inhibitors of angiogenesis in vitro and in vivo. *Journal of Biological Chemistry*.

[37] Murata T., He S., Hangai M. (2000). Peroxisome proliferator-activated receptor-gamma ligands inhibit choroidal neovascularization. *Investigative Ophthalmology & Visual Science*.

[38] Bishop-Bailey D., Hla T. (1999). Endothelial cell apoptosis induced by the peroxisome proliferator-activated receptor (PPAR) ligand 15-deoxy-delta 12, 14-prostaglandin J2. *Journal of Biological Chemistry*.

[39] Aljada A., O’Connor L., Fu Y.-Y., Mousa S. A. (2008). PPAR gamma ligands, rosiglitazone and pioglitazone, inhibit bFGF- and VEGF-mediated angiogenesis. *Angiogenesis*.

[40] Higuchi A., Ohashi K., Shibata R., Sono-Romanelli S., Walsh K., Ouchi N. (2010). Thiazolidinediones reduce pathological neovascularization in ischemic retina via an adiponectin-dependent mechanism. *Arteriosclerosis, Thrombosis, and Vascular Biology*.

[41] Stahl A., Sapieha P., Connor K. M. (2010). Short communication: PPAR gamma mediates a direct antiangiogenic effect of omega 3-PUFAs in proliferative retinopathy. *Circulation Research*.

[42] Gensch C., Clever Y. P., Werner C., Hanhoun M., Böhm M., Laufs U. (2007). The PPAR-*γ* agonist pioglitazone increases neoangiogenesis and prevents apoptosis of endothelial progenitor cells. *Atherosclerosis*.

[43] Nadra K., Quignodon L., Sardella C. (2010). PPAR*γ* in placental angiogenesis. *Endocrinology*.

[44] Kotlinowski J., Jozkowicz A. (2016). PPAR gamma and angiogenesis: endothelial cells perspective. *Journal of Diabetes Research*.

[45] Lecarpentier Y., Claes V., Vallée A., Hébert J. L. (2017). Thermodynamics in cancers: opposing interactions between PPAR gamma and the canonical WNT/beta-catenin pathway. *Clinical and Translational Medicine*.

[46] Stephen R. L., Gustafsson M. C. U., Jarvis M. (2004). Activation of peroxisome proliferator-activated receptor delta stimulates the proliferation of human breast and prostate cancer cell lines. *Cancer Research*.

[47] Liou J. Y., Lee S., Ghelani D., Matijevic-Aleksic N., Wu K. K. (2006). Protection of endothelial survival by peroxisome proliferator-activated Receptor-*δ* mediated 14-3-3 upregulation. *Arteriosclerosis, Thrombosis, and Vascular Biology*.

[48] Piqueras L., Reynolds A. R., Hodivala-Dilke K. M. (2007). Activation of PPAR*β*/*δ* induces endothelial cell proliferation and angiogenesis. *Arteriosclerosis, Thrombosis, and Vascular Biology*.

[49] Adamkiewicz J., Kaddatz K., Rieck M., Wilke B., Müller-Brüsselbach S., Müller R. (2007). Proteomic profile of mouse fibroblasts with a targeted disruption of the peroxisome proliferator activated receptor-beta/delta gene. *Proteomics*.

[50] Bohman S., Matsumoto T., Suh K. (2005). Proteomic analysis of vascular endothelial growth factor-induced endothelial cell differentiation reveals a role for chloride intracellular channel 4 (CLIC4) in tubular morphogenesis. *Journal of Biological Chemistry*.

[51] Kuppumbatti Y. S., Rexer B., Nakajo S., Nakaya K., Mira-y-Lopez R. (2001). CRBP suppresses breast cancer cell survival and anchorage-independent growth. *Oncogene*.

[52] Barak Y., Liao D., He W. Effects of peroxisome proliferator-activated receptor delta on placentation, adiposity, and colorectal cancer.

[53] Nadra K., Anghel S. I., Joye E. (2006). Differentiation of Trophoblast Giant Cells and Their Metabolic Functions Are Dependent on Peroxisome Proliferator-Activated Receptor *β*/*δ*. *Molecular and Cellular Biology*.

[54] Wieser F., Waite L., Depoix C., Taylor R. N. (2008). PPAR action in human placental development and pregnancy and its complications. *PPAR Research*.

[55] Luquet S., Lopez-Soriano J., Holst D. (2003). Peroxisome proliferator-activated receptor *δ* controls muscle development and oxydative capability. *The FASEB Journal*.

[56] Wang Y. X., Zhang C. L., Yu R. T. (2004). Regulation of muscle fiber type and running endurance by PPARdelta. *PLoS Biology*.

[57] Narkar V. A., Downes M., Yu R. T. (2008). AMPK and PPAR*δ* Agonists Are Exercise Mimetics. *Cell*.

[58] Gaudel C., Schwartz C., Giordano C., Abumrad N. A., Grimaldi P. A. (2008). Pharmacological activation of PPARbeta promotes rapid and calcineurin-dependent fiber remodeling and angiogenesis in mouse skeletal muscle. *American Journal of Physiology-Endocrinology and Metabolism*.

[59] Capozzi M. E., McCollum G. W., Savage S. R., Penn J. S. (2013). Peroxisome proliferator-activated receptor-*β*/*δ* regulates angiogenic cell behaviors and oxygen-induced retinopathy. *Investigative Opthalmology & Visual Science*.

[60] Choudhary M., Ding J.-d., Qi X. (2016). PPAR*β*/*δ* selectively regulates phenotypic features of age-related macular degeneration. *Aging*.

[61] Yasumatsu R., Nakashima T., Masuda M. (2004). Effects of the angiotensin-I converting enzyme inhibitor perindopril on tumor growth and angiogenesis in head and neck squamous cell carcinoma cells. *Journal of Cancer Research and Clinical Oncology*.

[62] Volpert O. V., Ward W. F., Lingen M. W. (1996). Captopril inhibits angiogenesis and slows the growth of experimental tumors in rats. *Journal of Clinical Investigation*.

[63] Dueñas-González A., García-López P., Herrera L., Medina-Franco J., González-Fierro A., Candelaria M. (2008). The prince and the pauper. A tale of anticancer targeted agents. *Molecular Cancer*.

[64] Kang E. S., Hwang J. S., Lee W. J. (2019). Ligand-activated PPAR*δ* inhibits angiotensin II-stimulated hypertrophy of vascular smooth muscle cells by targeting ROS. *PLOS ONE*.

[65] Takata Y., Liu J., Yin F. PPARdelta-mediated antiinflammatory mechanisms inhibit angiotensin II-accelerated atherosclerosis.

[66] Vallée A., Lévy B. L., Blacher J. (2018). Interplay between the renin-angiotensin system, the canonical WNT/*β*-catenin pathway and PPAR*γ* in hypertension. *Current Hypertension Reports*.

[67] Han J. K., Lee H. S., Yang H. M. (2008). Peroxisome proliferator-activated receptor-delta agonist enhances vasculogenesis by regulating endothelial progenitor cells through genomic and nongenomic activations of the phosphatidylinositol 3-kinase/Akt pathway. *Circulation*.

[68] He T., Lu T., d'Uscio L. V., Lam C. F., Lee H. C., Katusic Z. S. (2008). Angiogenic function of prostacyclin biosynthesis in human endothelial progenitor cells. *Circulation Research*.

[69] Han J. K., Kim H. L., Jeon K. H. (2013). Peroxisome proliferator-activated receptor-*δ* activates endothelial progenitor cells to induce angio-myogenesis through matrix metallo-proteinase-9-mediated insulin-like growth factor-1 paracrine networks. *European Heart Journal*.

[70] Gupta R. A., Tan J., Krause W. F. Prostacyclin-mediated activation of peroxisome proliferator-activated receptor delta in colorectal cancer.

[71] Pedchenko T. V., Gonzalez A. L., Wang D., DuBois R. N., Massion P. P. (2008). Peroxisome proliferator-activated receptor beta/delta expression and activation in lung cancer. *American Journal of Respiratory Cell and Molecular Biology*.

[72] Yuan H., Lu J., Xiao J. (2013). PPAR*δ* induces estrogen receptor-positive mammary neoplasia through an inflammatory and metabolic phenotype linked to mTOR activation. *Cancer Research*.

[73] Zuo X., Deguchi Y., Xu W. (2019). PPARD and interferon gamma promote transformation of gastric progenitor cells and tumorigenesis in mice. *Gastroenterology*.

[74] Peters J. M., Foreman J. E., Gonzalez F. J. (2011). Dissecting the role of peroxisome proliferator-activated receptor-*β*/*δ* (PPAR*β*/*δ*) in colon, breast, and lung carcinogenesis. *Cancer and Metastasis Reviews*.

[75] Peters J. M., Shah Y. M., Gonzalez F. J. (2012). The role of peroxisome proliferator-activated receptors in carcinogenesis and chemoprevention. *Nature Reviews Cancer*.

[76] Peters J. M., Gonzalez F. J., Müller R. (2015). Establishing the role of PPAR*β*/*δ* in carcinogenesis. *Trends in Endocrinology & Metabolism*.

[77] Wagner N., Wagner K. D. (2020). PPAR beta/delta and the hallmarks of cancer. *Cells*.

[78] Bishop-Bailey D., Swales K. E. (2008). The role of PPARs in the endothelium: implications for cancer therapy. *PPAR Research*.

[79] Bishop-Bailey D. (2008). A Role for PPAR *β* / *δ* in Ocular Angiogenesis. *PPAR Research*.

[80] Bishop-Bailey D. (2011). PPARs and angiogenesis. *Biochemical Society Transactions*.

[81] Jeong E., Koo J. E., Yeon S. H., Kwak M. K., Hwang D. H., Lee J. Y. (2014). PPAR*δ* deficiency disrupts hypoxia-mediated tumorigenic potential of colon cancer cells. *Molecular Carcinogenesis*.

[82] Condeelis J., Pollard J. W. (2006). Macrophages: obligate partners for tumor cell migration, invasion, and metastasis. *Cell*.

[83] Schmid M. C., Varner J. A. (2010). Myeloid cells in the tumor microenvironment: modulation of tumor angiogenesis and tumor inflammation. *Journal of Oncology*.

[84] Park J., Lee S. E., Hur J. (2015). M-CSF from cancer cells induces fatty acid synthase and PPAR*β*/*δ* activation in tumor myeloid cells, leading to tumor progression. *Cell Reports*.

[85] Tsai Y. C., Weissman A. M. (2010). The unfolded protein response, degradation from endoplasmic reticulum and cancer. *Genes Cancer*.

[86] Salvadó L., Barroso E., Gómez-Foix A. M. (2014). PPAR*β*/*δ* prevents endoplasmic reticulum stress-associated inflammation and insulin resistance in skeletal muscle cells through an AMPK-dependent mechanism. *Diabetologia*.

[87] Palomer X., Capdevila-Busquets E., Botteri G. (2014). PPAR*β*/*δ* attenuates palmitate-induced endoplasmic reticulum stress and induces autophagic markers in human cardiac cells. *International Journal of Cardiology*.

[88] Zhu B., Ferry C. H., Markell L. K. (2014). The nuclear receptor peroxisome proliferator-activated receptor-*β*/*δ* (PPAR*β*/*δ*) promotes oncogene-induced cellular senescence through repression of endoplasmic reticulum stress. *Journal of Biological Chemistry*.

[89] Zhu B., Ferry C. H., Blazanin N. (2014). PPAR*β*/*δ* promotes HRAS-induced senescence and tumor suppression by potentiating p-ERK and repressing p-AKT signaling. *Oncogene*.

[90] Grosse L., Wagner N., Emelyanov A. (2020). Defined p16^High^ Senescent Cell Types Are Indispensable for Mouse Healthspan. *Cell Metabolism*.

[91] Wagner K. D., Vukolic A., Baudouy D., Michiels J. F., Wagner N. (2018). Erratum to “Inducible conditional vascular-specific overexpression of peroxisome proliferator-activated receptor beta/delta leads to rapid cardiac hypertrophy”. *PPAR Research*.

[92] Wagner K. D., Vukolic A., Baudouy D., Michiels J. F., Wagner N. (2016). Inducible conditional vascular-specific overexpression of peroxisome proliferator-activated receptor beta/delta leads to rapid cardiac hypertrophy. *PPAR Research*.

[93] Gately S., Li W. W. (2004). Multiple roles of COX-2 in tumor angiogenesis: a target for antiangiogenic therapy. *Seminars in Oncology*.

[94] Nagy J. A., Dvorak A. M., Dvorak H. F. (2007). VEGF-A and the induction of pathological angiogenesis. *Annual Review of Pathology: Mechanisms of Disease*.

[95] Gerhardt H., Golding M., Fruttiger M. (2003). VEGF guides angiogenic sprouting utilizing endothelial tip cell filopodia. *Journal of Cell Biology*.

[96] Hellström M., Phng L. K., Gerhardt H. (2014). VEGF and Notch Signaling. *Cell Adhesion & Migration*.

[97] Bergers G., Brekken R., McMahon G. (2000). Matrix metalloproteinase-9 triggers the angiogenic switch during carcinogenesis. *Nature Cell Biology*.

[98] Deryugina E. I., Quigley J. P. (2015). Tumor angiogenesis: MMP-mediated induction of intravasation- and metastasis- sustaining neovasculature. *Matrix Biology*.

[99] Park H.-Y., Kwon H. M., Lim H. J. (2001). Potential role of leptin in angiogenesis: leptin induces endothelial cell proliferation and expression of matrix metalloproteinases in vivo and in vitro. *Experimental & Molecular Medicine*.

[100] Garonna E., Botham K. M., Birdsey G. M., Randi A. M., Gonzalez-Perez R. R., Wheeler-Jones C. P. D. (2011). Vascular endothelial growth factor receptor-2 couples cyclo-oxygenase-2 with pro-angiogenic actions of leptin on human endothelial cells. *PLoS ONE*.

[101] Lanier V., Gillespie C., Leffers M. (2016). Leptin-induced transphosphorylation of vascular endothelial growth factor receptor increases Notch and stimulates endothelial cell angiogenic transformation. *The International Journal of Biochemistry & Cell Biology*.

[102] Wang D., Fu L., Ning W. Peroxisome proliferator-activated receptor *δ* promotes colonic inflammation and tumor growth.

[103] Wang D., DuBois R. N. (2010). Therapeutic potential of peroxisome proliferator-activated receptors in chronic inflammation and colorectal cancer. *Gastroenterology Clinics of North America*.

[104] Glinghammar B., Skogsberg J., Hamsten A., Ehrenborg E. (2003). PPAR*δ* activation induces COX-2 gene expression and cell proliferation in human hepatocellular carcinoma cells. *Biochemical and Biophysical Research Communications*.

[105] Wang D., Wang H., Guo Y. Crosstalk between peroxisome proliferator-activated receptor delta and VEGF stimulates cancer progression.

[106] Zuo X., Peng Z., Moussalli M. J. (2009). Targeted genetic disruption of peroxisome proliferator-activated receptor-delta and colonic tumorigenesis. *JNCI: Journal of the National Cancer Institute*.

[107] Wagner K. D., Benchetrit M., Bianchini L., Michiels J. F., Wagner N. (2011). Peroxisome proliferator-activated receptor *β*/*δ* (PPAR*β*/*δ*) is highly expressed in liposarcoma and promotes migration and proliferation. *The Journal of Pathology*.

[108] Qian B. Z., Pollard J. W. (2010). Macrophage diversity enhances tumor progression and metastasis. *Cell*.

[109] Coffelt S. B., Tal A. O., Scholz A. (2010). Angiopoietin-2 regulates gene expression in TIE2-expressing monocytes and augments their inherent proangiogenic functions. *Cancer Research*.

[110] Roussos E. T., Condeelis J. S., Patsialou A. (2011). Chemotaxis in cancer. *Nature Reviews Cancer*.

[111] Põld M., Zhu L. X., Sharma S. (2004). Cyclooxygenase-2-dependent expression of angiogenic CXC chemokines ENA-78/CXC ligand (CXCL) 5 and interleukin-8/CXCL8 in human non-small cell lung cancer. *Cancer Research*.

[112] Tazzyman S., Lewis C. E., Murdoch C. (2009). Neutrophils: key mediators of tumour angiogenesis. *International Journal of Experimental Pathology*.

[113] Nozawa H., Chiu C., Hanahan D. Infiltrating neutrophils mediate the initial angiogenic switch in a mouse model of multistage carcinogenesis.

[114] Deryugina E. I., Zajac E., Juncker-Jensen A., Kupriyanova T. A., Welter L., Quigley J. P. (2014). Tissue-Infiltrating Neutrophils Constitute the Major _In Vivo_ Source of Angiogenesis-Inducing MMP-9 in the Tumor Microenvironment. *Neoplasia*.

[115] Stamatovic S. M., Keep R. F., Mostarica-Stojkovic M., Andjelkovic A. V. (2006). CCL2 regulates angiogenesis via activation of Ets-1 transcription factor. *The Journal of Immunology*.

[116] Ehling J., Bartneck M., Wei X. (2014). CCL2-dependent infiltrating macrophages promote angiogenesis in progressive liver fibrosis. *Gut*.

[117] Roblek M., Protsyuk D., Becker P. F. (2019). CCL2 is a vascular permeability factor inducing CCR2-dependent endothelial retraction during lung metastasis. *Molecular Cancer Research*.

[118] Joyce J. A., Pollard J. W. (2009). Microenvironmental regulation of metastasis. *Nature Reviews Cancer*.

[119] Danese S., Mantovani A. (2010). Inflammatory bowel disease and intestinal cancer: a paradigm of the Yin-Yang interplay between inflammation and cancer. *Oncogene*.

[120] Erreni M., Mantovani A., Allavena P. (2011). Tumor-associated macrophages (TAM) and inflammation in colorectal cancer. *Cancer Microenviron*.

[121] Breier G., Blum S., Peli J. (2002). Transforming growth factor-beta and Ras regulate the VEGF/VEGF-receptor system during tumor angiogenesis. *International Journal of Cancer*.

[122] Kuwano T., Nakao S., Yamamoto H. (2003). Cyclooxygenase 2 is a key enzyme for inflammatory cytokine-induced angiogenesis. *The FASEB Journal*.

[123] Nakao S., Kuwano T., Tsutsumi-Miyahara C. (2005). Infiltration of COX-2-expressing macrophages is a prerequisite for IL-1 beta-induced neovascularization and tumor growth. *Journal of Clinical Investigation*.

[124] Amano K., Okigaki M., Adachi Y. (2004). Mechanism for IL-1*β*-mediated neovascularization unmasked by IL-1*β* knock-out mice. *Journal of Molecular and Cellular Cardiology*.

[125] Voronov E., Carmi Y., Apte R. N. (2014). The role IL-1 in tumor-mediated angiogenesis. *Frontiers in Physiology*.

[126] Fan Y., Ye J., Shen F. (2007). Interleukin-6 stimulates circulating blood-derived endothelial progenitor cell Angiogenesisin vitro. *Journal of Cerebral Blood Flow & Metabolism*.

[127] Nilsson M. B., Langley R. R., Fidler I. J. (2005). Interleukin-6, secreted by human ovarian carcinoma cells, is a potent proangiogenic cytokine. *Cancer Research*.

[128] Gopinathan G., Milagre C., Pearce O. M. T. (2015). Interleukin-6 stimulates defective angiogenesis. *Cancer Research*.

[129] Wagner K. D., Cherfils-Vicini J., Hosen N. (2014). The Wilms' tumour suppressor Wt1 is a major regulator of tumour angiogenesis and progression. *Nature Communications*.

[130] Oettgen P. (2006). Regulation of vascular inflammation and remodeling by ETS factors. *Circulation Research*.

[131] Singh S., Barrett J., Sakata K., Tozer R. G., Singh G. (2002). ETS Proteins and MMPs: Partners in Invasion and Metastasis. *Current Drug Targets*.

[132] Vetter M., Blumenthal S. G., Lindemann R. K. (2005). Ets1 is an effector of protein kinase C _*α*_ in cancer cells. *Oncogene*.

[133] Nakamura Y., Esnault S., Maeda T., Kelly E. A. B., Malter J. S., Jarjour N. N. (2004). Ets-1 Regulates TNF-*α*-Induced Matrix Metalloproteinase-9 and Tenascin Expression in Primary Bronchial Fibroblasts. *The Journal of Immunology*.

[134] Wernert N. (2011). Identification of ETS-1 target genes in human fibroblasts. *International Journal of Oncology*.

[135] Erez N., Truitt M., Olson P., Arron S. T., Hanahan D. (2010). Cancer-Associated Fibroblasts Are Activated in Incipient Neoplasia to Orchestrate Tumor-Promoting Inflammation in an NF-*κ*B-Dependent Manner. *Cancer Cell*.

[136] Giannoni E., Bianchini F., Masieri L. (2010). Reciprocal activation of prostate cancer cells and cancer-associated fibroblasts stimulates epithelial-mesenchymal transition and cancer stemness. *Cancer Research*.

[137] Kalluri R. (2016). The biology and function of fibroblasts in cancer. *Nature Reviews Cancer*.

[138] Tan E. H. P., Sng M. K., How I. S. B. (2018). ROS release by _PPAR_ *β*/*δ*-null fibroblasts reduces tumor load through epithelial antioxidant response. *Oncogene*.

[139] Hashiya N., Jo N., Aoki M. (2004). In vivo evidence of angiogenesis induced by transcription factor Ets-1: Ets-1 is located upstream of angiogenesis cascade. *Circulation*.

[140] Dutta D., Ray S., Vivian J. L., Paul S. (2008). Activation of the VEGFR1 chromatin domain: an angiogenic signal-ETS1/HIF-2alpha regulatory axis. *Journal of Biological Chemistry*.

[141] Elvert G., Kappel A., Heidenreich R. (2003). Cooperative interaction of hypoxia-inducible factor-2alpha (HIF-2alpha) and Ets-1 in the transcriptional activation of vascular endothelial growth factor receptor-2 (Flk-1). *J Biol Chem*.

[142] Watanabe D., Takagi H., Suzuma K. (2004). Transcription factor Ets-1 mediates ischemia- and vascular endothelial growth factor-dependent retinal neovascularization. *The American Journal of Pathology*.

[143] Lavenburg K. R., Ivey J., Hsu T., Muise-Helmericks R. C. (2003). Coordinated functions of Akt/PKB and ETS1 in tubule formation. *The FASEB Journal*.

[144] Wagner N., Michiels J. F., Schedl A., Wagner K. D. (2008). The Wilms' tumour suppressor WT1 is involved in endothelial cell proliferation and migration: expression in tumour vessels _in vivo_. *Oncogene*.

[145] Darby I. A., Bisucci T., Raghoenath S., Olsson J., Muscat G. E. O., Koopman P. (2001). _Sox18_ Is Transiently Expressed during Angiogenesis in Granulation Tissue of Skin Wounds with an Identical Expression Pattern to _Flk-1_ mRNA. *Laboratory Investigation*.

[146] Duong T., Proulx S. T., Luciani P. (2012). Genetic ablation of SOX18 function suppresses tumor lymphangiogenesis and metastasis of melanoma in mice. *Cancer Research*.

[147] Donovan P., Patel J., Dight J. (2019). Endovascular progenitors infiltrate melanomas and differentiate towards a variety of vascular beds promoting tumor metastasis. *Nature Communications*.

[148] Michiels J. F., Perrin C., Leccia N., Massi D., Grimaldi P., Wagner N. (2010). PPARbeta activation inhibits melanoma cell proliferation involving repression of the Wilms’ tumour suppressor WT1. *Pflügers Archiv - European Journal of Physiology*.

[149] Wang D., Fu L., Wei J., Xiong Y., DuBois R. N. (2019). PPAR*δ* mediates the effect of dietary fat in promoting colorectal cancer metastasis. *Cancer Research*.

[150] Yu J., Vodyanik M. A., Smuga-Otto K. (2007). Induced pluripotent stem cell lines derived from human somatic cells. *Science*.

[151] Shan J., Shen J., Liu L. (2012). Nanog regulates self-renewal of cancer stem cells through the insulin-like growth factor pathway in human hepatocellular carcinoma. *Hepatology*.

[152] Foster B., Zaidi D., Young T., Mobley M., Kerr B. (2018). CD117/c-kit in Cancer Stem Cell-Mediated Progression and Therapeutic Resistance. *Biomedicines*.

[153] Broudy V. C., Kovach N. L., Bennett L. G., Lin N., Jacobsen F. W., Kidd P. G. (1994). Human umbilical vein endothelial cells display high-affinity c-kit receptors and produce a soluble form of the c-kit receptor. *Blood*.

[154] Matsui J., Wakabayashi T., Asada M., Yoshimatsu K., Okada M. (2004). Stem cell factor/c-kit signaling promotes the survival, migration, and capillary tube formation of human umbilical vein endothelial cells. *Journal of Biological Chemistry*.

[155] Sihto H., Tynninen O., Bützow R., Saarialho-Kere U., Joensuu H. (2007). Endothelial cell KIT expression in human tumours. *The Journal of Pathology*.

[156] Puputti M., Tynninen O., Pernilä P. (2010). Expression of KIT receptor tyrosine kinase in endothelial cells of juvenile brain tumors. *Brain Pathology*.

[157] Fang S., Wei J., Pentinmikko N., Leinonen H., Salven P. (2012). Generation of functional blood vessels from a single c-kit+ adult vascular endothelial stem cell. *PLoS Biology*.

[158] Qin S., Li A., Yi M., Yu S., Zhang M., Wu K. (2019). Recent advances on anti-angiogenesis receptor tyrosine kinase inhibitors in cancer therapy. *Journal of Hematology & Oncology*.

[159] Jin T., Nakatani H., Taguchi T. (2006). STI571 (Glivec) suppresses the expression of vascular endothelial growth factor in the gastrointestinal stromal tumor cell line, GIST-T1. *World Journal of Gastroenterology*.

[160] Litz J., Krystal G. W. (2006). Imatinib inhibits c-Kit-induced hypoxia-inducible factor-1alpha activity and vascular endothelial growth factor expression in small cell lung cancer cells. *Molecular Cancer Therapeutics*.

[161] Kim K. L., Seo S., Kim J. T. (2019). SCF (stem cell factor) and cKIT modulate pathological ocular neovascularization. *Arteriosclerosis, Thrombosis, and Vascular Biology*.

[162] Bjarnegård M., Enge M., Norlin J. (2004). Endothelium-specific ablation of PDGFB leads to pericyte loss and glomerular, cardiac and placental abnormalities. *Development*.

[163] Magnusson P. U., Looman C., Ahgren A., Wu Y., Claesson-Welsh L., Heuchel R. L. (2007). Platelet-derived growth factor Receptor-*β* constitutive activity promotes angiogenesis in vivo and in vitro. *Arteriosclerosis, Thrombosis, and Vascular Biology*.

[164] von Ballmoos M. W., Yang Z., Völzmann J., Baumgartner I., Kalka C., Di Santo S. (2010). Endothelial Progenitor Cells Induce a Phenotype Shift in Differentiated Endothelial Cells towards PDGF/PDGFR*β* Axis-Mediated Angiogenesis. *PLoS ONE*.

[165] Wang H., Yin Y., Li W. (2012). Over-expression of PDGFR-*β* promotes PDGF-induced proliferation, migration, and angiogenesis of EPCs through PI3K/Akt signaling pathway. *PLoS ONE*.

[166] Lindahl P., Johansson B. R., Levéen P., Betsholtz C. (1997). Pericyte loss and microaneurysm formation in PDGF-B-deficient mice. *Science*.

[167] Hellström M., Kalén M., Lindahl P., Abramsson A., Betsholtz C. (1999). Role of PDGF-B and PDGFR-beta in recruitment of vascular smooth muscle cells and pericytes during embryonic blood vessel formation in the mouse. *Development*.

[168] Wang Y., Jin Y., Mäe M. A. (2017). Smooth muscle cell recruitment to lymphatic vessels requires PDGFB and impacts vessel size but not identity. *Development*.

[169] Nissen L. J., Cao R., Hedlund E. M. (2007). Angiogenic factors FGF2 and PDGF-BB synergistically promote murine tumor neovascularization and metastasis. *Journal of Clinical Investigation*.

[170] Zhang J., Cao R., Zhang Y., Jia T., Cao Y., Wahlberg E. (2008). Differential roles of PDGFR-alpha and PDGFR-beta in angiogenesis and vessel stability. *The FASEB Journal*.

[171] Gialeli C., Nikitovic D., Kletsas D., Theocharis A., Tzanakakis G., Karamanos N. (2014). PDGF/PDGFR signaling and targeting in cancer growth and progression: focus on tumor microenvironment and cancer-associated fibroblasts. *Current Pharmaceutical Design*.

[172] Rizvi S., Mertens J. C., Bronk S. F. (2014). Platelet-derived growth factor primes cancer-associated fibroblasts for apoptosis. *Journal of Biological Chemistry*.

[173] Chu T. Y., Yang J. T., Huang T. H., Liu H. W. (2014). Crosstalk with cancer-associated fibroblasts increases the growth and radiation survival of cervical cancer cells. *Radiation Research*.

[174] Yang Y., Andersson P., Hosaka K. (2016). The PDGF-BB-SOX7 axis-modulated IL-33 in pericytes and stromal cells promotes metastasis through tumour-associated macrophages. *Nature Communications*.

[175] Tsioumpekou M., Cunha S. I., Ma H. (2020). Specific targeting of PDGFR*β* in the stroma inhibits growth and angiogenesis in tumors with high PDGF-BB expression. *Theranostics*.

[176] El Maï M., Wagner K.-D., Michiels J.-F. (2014). The Telomeric Protein TRF2 Regulates Angiogenesis by Binding and Activating the PDGFR*β* Promoter. *Cell Reports*.

[177] Palmer C. N. A., Hsu M.-H., Griffin K. J., Johnson E. F. (1995). Novel sequence determinants in peroxisome proliferator signaling. *Journal of Biological Chemistry*.

[178] Gealekman O., Burkart A., Chouinard M., Nicoloro S. M., Straubhaar J., Corvera S. (2008). Enhanced angiogenesis in obesity and in response to PPARgamma activators through adipocyte VEGF and ANGPTL4 production. *American Journal of Physiology-Endocrinology and Metabolism*.

[179] Le Jan S., Amy C., Cazes A. (2003). Angiopoietin-like 4 is a proangiogenic factor produced during ischemia and in conventional renal cell carcinoma. *The American Journal of Pathology*.

[180] Adhikary T., Brandt D. T., Kaddatz K. (2013). Inverse PPAR*β*/*δ* agonists suppress oncogenic signaling to the _ANGPTL4_ gene and inhibit cancer cell invasion. *Oncogene*.

[181] Tan M. J., Teo Z., Sng M. K., Zhu P., Tan N. S. (2012). Emerging roles of angiopoietin-like 4 in human cancer. *Molecular Cancer Research*.

[182] Mandard S., Zandbergen F., van Straten E. (2006). The fasting-induced adipose factor/angiopoietin-like protein 4 is physically associated with lipoproteins and governs plasma lipid levels and adiposity. *Journal of Biological Chemistry*.

[183] Staiger H., Haas C., Machann J. (2009). Muscle-derived angiopoietin-like protein 4 is induced by fatty acids via peroxisome proliferator-activated receptor (PPAR)-delta and is of metabolic relevance in humans. *Diabetes*.

[184] Inoue T., Kohro T., Tanaka T. (2014). Cross-enhancement of ANGPTL4 transcription by HIF1 alpha and PPAR beta/delta is the result of the conformational proximity of two response elements. *Genome Biology*.

[185] Sun Y., Daemen A., Hatzivassiliou G. (2014). Metabolic and transcriptional profiling reveals pyruvate dehydrogenase kinase 4 as a mediator of epithelial-mesenchymal transition and drug resistance in tumor cells. *Cancer & Metabolism*.

[186] Menendez J. A., Lupu R. (2007). Fatty acid synthase and the lipogenic phenotype in cancer pathogenesis. *Nature Reviews Cancer*.

[187] Zaidi N., Lupien L., Kuemmerle N. B., Kinlaw W. B., Swinnen J. V., Smans K. (2013). Lipogenesis and lipolysis: the pathways exploited by the cancer cells to acquire fatty acids. *Progress in Lipid Research*.

[188] Guda M. R., Asuthkar S., Labak C. M. (2018). Targeting PDK4 inhibits breast cancer metabolism. *American Journal of Cancer Research*.

[189] Degenhardt T., Saramäki A., Malinen M. (2007). Three Members of the Human Pyruvate Dehydrogenase Kinase Gene Family Are Direct Targets of the Peroxisome Proliferator-activated Receptor *β*/*δ*. *Journal of Molecular Biology*.

[190] Wolfrum C. (2007). Lipid sensing and lipid sensors. *Cellular and Molecular Life Sciences*.

[191] De Santis M. L., Hammamieh R., Das R., Jett M. (2004). Adipocyte-fatty acid binding protein induces apoptosis in DU145 prostate cancer cells. *Journal of Experimental Therapeutics and Oncology*.

[192] Shin J., Li B., Davis M. E., Suh Y., Lee K. (2009). Comparative analysis of fatty acid-binding protein 4 promoters: conservation of peroxisome proliferator-activated receptor binding sites1. *Journal of Animal Science*.

[193] Kavanagh E., Joseph B. (2011). The hallmarks of CDKN1C (p57, KIP2) in cancer. *Biochimica et Biophysica Acta*.

[194] Montagner A., Delgado M. B., Tallichet-Blanc C. (2013). Src is activated by the nuclear receptor peroxisome proliferator-activated receptor *β*/*δ* in ultraviolet radiation-induced skin cancer. *EMBO Molecular Medicine*.

[195] Han J. K., Kim B. K., Won J. Y. (2016). Interaction between platelets and endothelial progenitor cells via LPA-Edg-2 axis is augmented by PPAR-*δ* activation. *Journal of Molecular and Cellular Cardiology*.

[196] Wilhelm K., Happel K., Eelen G. (2016). FOXO1 couples metabolic activity and growth state in the vascular endothelium. *Nature*.

[197] Jeon H. H., Yu Q., Lu Y. (2018). FOXO1 regulates VEGFA expression and promotes angiogenesis in healing wounds. *The Journal of Pathology*.

[198] Kim Y. H., Choi J., Yang M. J. (2019). A MST1-FOXO1 cascade establishes endothelial tip cell polarity and facilitates sprouting angiogenesis. *Nature Communications*.

[199] Nahlé Z., Hsieh M., Pietka T. (2008). CD36-dependent regulation of muscle FoxO1 and PDK4 in the PPAR*δ*/*β*-mediated adaptation to metabolic stress. *Journal of Biological Chemistry*.

[200] Zhao F.-Q., Keating A. (2007). Functional properties and genomics of glucose transporters. *Current Genomics*.

[201] Young C. D., Lewis A. S., Rudolph M. C. (2011). Modulation of glucose transporter 1 (GLUT1) expression levels alters mouse mammary tumor cell growth in vitro and in vivo. *PLoS ONE*.

[202] Zhang W., Xu Y., Xu Q., Shi H., Shi J., Hou Y. (2017). PPAR*δ* promotes tumor progression via activation of Glut 1 and SLC1-A5 transcription. *Carcinogenesis*.

[203] Nicklin P., Bergman P., Zhang B. (2009). Bidirectional transport of amino acids regulates mTOR and autophagy. *Cell*.

[204] Jeon Y. J., Khelifa S., Ratnikov B. (2015). Regulation of glutamine carrier proteins by RNF5 determines breast cancer response to ER stress-inducing chemotherapies. *Cancer Cell*.

